# Cleaning Effectiveness of Three NiTi Rotary Instruments: A Focus on Biomaterial Properties

**DOI:** 10.3390/jfb6010066

**Published:** 2015-02-16

**Authors:** Claudio Poggio, Alberto Dagna, Marco Chiesa, Riccardo Beltrami, Stefano Bianchi

**Affiliations:** 1 Department of Clinical-Surgical, Diagnostic and Pediatric Sciences, University of Pavia, Piazzale Golgi 3, 27100 Pavia, Italy; E-Mails: claudio.poggio@unipv.it (C.P.); alberto.dagna@unipv.it (A.D.); chiesamarco@hotmail.com (M.C.); stefano.bianchi@unipv.it (S.B.); 2 Department of Brain and Behavioural Sciences—Section of Statistics, University of Pavia, Via Bassi 21, 27100 Pavia, Italy

**Keywords:** debris, NiTi instruments, SEM, smear layer

## Abstract

Nickel-titanium (NiTi) instruments are commonly used for shaping the root canal system in endodontic practice. They are more flexible and have better cutting efficiency than conventional stainless steel files. The superelasticity of NiTi rotary files allows the clinicians to produce the desirable tapered root canal form with a reduced tendency to canal transportation and instrument fracture. HyFlex CM instruments are new NiTi rotary instruments with shape memory produced by an innovative methodology (patent pending) that uses a complex heating and cooling treatment that controls the material’s memory. The aim of the present study was to compare the cleaning efficacy of two conventional (Mtwo, Revo-S) Ni-Ti rotary instruments with HyFlex CM. 30 single-rooted freshly extracted teeth were divided into three groups. Root canals were shaped with three NiTi instruments (Mtwo, Revo-S and HyFlex CM) using 5.25% NaOCl and 17% EDTA solutions. Specimens were fractured longitudinally and prepared for SEM analysis at standard magnification of 1000×. The presence/absence of debris smear layer and the presence/absence of smear layer at coronal, middle, and apical third of each canal were evaluated using a 5-step scale for scores. Numeric data were analyzed using Kruskall-Wallis and Mann-Whitney *U* statistical tests and significance was predetermined at *P* < 0.05. This study revealed significant differences among the various groups. Despite some minor differences, all instruments removed smear layer and debris produced during instrumentation. HyFlex CM seem to be not so effective in promoting cleanliness of root canal walls and in removing smear layer from dentine if compared to Mtwo and Revo-S.

## 1. Introduction

Root canal treatment is based on cleaning, shaping and sealing the root canal system [1]. Its main objective is the elimination of microorganisms from the root canals and the prevention of recontamination after filling [2,3,4,5]. Irrigating solutions facilitates the disinfection and the debridement of the root canal, so they are considered to be essential for successful endodontic treatment [6,7,8,9,10]. Instruments alone cannot effectively eliminate bacteria from the root canal system [11] and modern rotary instrumentation techniques produce a large quantity of smear layer that covers root canal walls. In the last decade many nickel-titanium (NiTi) rotary instruments have been introduced. All NiTi rotating instruments have been shown to produce moderate to heavy smear layer that need to be removed with the use of chemical solutions [12,13]. The chelating agents like ethylenediaminetetraacetic acid (EDTA) are currently used to remove the smear layer formed during preparation of the root canals [14]. The association of EDTA and NaOCl solutions is the gold standard in chemo-mechanical preparation of the root canals [15,16]. EDTA acts upon the inorganic components of the smear layer and decalcifies the peri- and intertubular dentine and leaves the collagen exposed. Subsequently, the use of NaOCl dissolves the collagen, cleaning the dentinal walls [14]. Combined use of irrigating solutions and rotary instruments decreases bacterial counts in the root canal when compared to standard instrumentation alone [17]. Several SEM studies revealed that rotating files associated to EDTA and NaOCl irrigation leave dentine surfaces substantially free from smear layer [18,19,20]. The combination of NaOCl and EDTA favorites the removal of smear layer and the removal of a great portion of circumferential dentinal collagen and mineralized dentine from the surfaces of tubules, as confirmed by Foschi *et al.* [18]. This means that absence of smear layer and presence of clean dentinal walls provide a reduction in bacterial count. Today is well known that mechanical NiTi instrumentation in combination with chemical cleaning greatly reduces the microorganisms remaining in the root canal system [21,22,23]. Total removal of smear layer facilitates the diffusion of the irrigants and the medications to the root canal system [24] and then improves the adaptation of the filling materials to the root canal dentine, reducing apical and coronal microleakage of the root canal filling materials [25]. The purpose of the present study was to investigate the cleaning efficacy of three NiTi rotary instruments: Mtwo, Revo-S and HyFlex CM. The amount of debris and the morphology of smear layer were parameters for the evaluation of the cleanliness of root canals. Higher presence of debris and smear layer is indicated by higher score values. The null hypothesis of the study is that there is no significant difference in debris scores and smear layer scores between the three systems.

## 2. Results and Discussion

**Figure 1:**
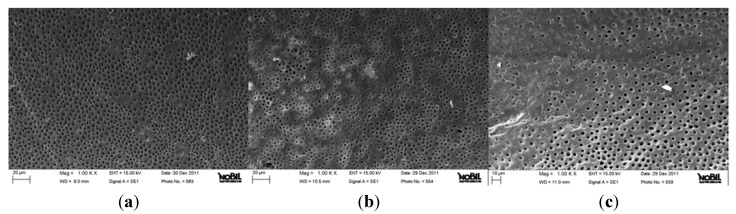
Representative samples of scanning electron micrographs of the root canal dentin surface instrumented with Mtwo (group A) at coronal (**a**), middle (**b**) and apical (**c**) third of the root (1000×). Dentinal tubules are clearly visible. Small debris particles are present (Smear layer and debris scores = 1).

**Figure 2:**
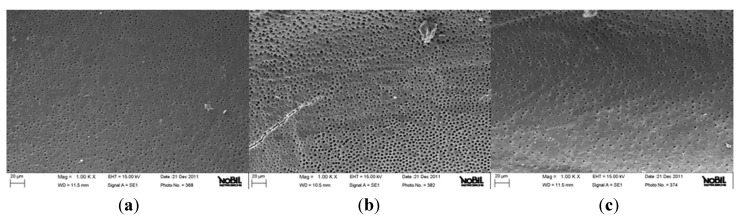
Representative samples of scanning electron micrographs of the root canal dentin surface instrumented with Revo-S (group B) at coronal (**a**), middle (**b**) and apical (**c**) third of the root (1000×). Dentinal tubules are clearly visible. Small debris particles are present (Smear layer and debris scores = 1).

**Figure 3:**
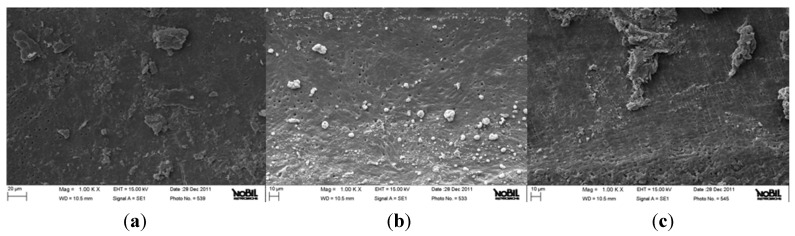
Representative samples of scanning electron micrographs of the root canal dentin surface instrumented with HyFlex CM (group C) at coronal (**a**), middle (**b**) and apical (**c**) third of the root (1000×). Few dentinal tubules are open. Homogeneous smear layer covers root canal wall (Smear layer score = 3). Many agglomerations of debris cover less than 50% of the surface of the root wall (Debris score = 3).

The mean amounts of debris and smear layer scores of the three groups are reported in Table 1, Table 2, Table 3 and Table 4. Kruskall-Wallis test showed the presence of significant among the different groups (*P* < 0.05). Mann-Whitney *U* test showed no significant difference in debris scores between Mtwo and Revo-S groups when comparing coronal, middle and apical thirds (*P* > 0.05). HyFlex CM groups showed significantly higher scores than other groups tested (*P* < 0.05). Moreover Mann-Whitney *U* test showed no significant difference in smear layer scores between Mtwo and Revo-S groups when comparing coronal, middle and apical thirds (*P* > 0.05). HyFlex CM groups showed significantly higher scores than other groups tested (*P* < 0.05). Figure 1, Figure 2 and Figure 3 show representative samples of SEM micrographs (1000×) of the root canal dentin surface of groups A, B and C.

**Table 1:** Average score of the debris for the coronal, middle and apical third of the canals (Values with the same superscript letters were not statistically different at * *P* = 0.05).

Group	Coronal	Middle	Apical	Overall
Mtwo	1.33 ^a^	1.40 ^a^	1.53 ^a^	1.42 ^a^
Revo-S	1.33 ^a^	1.53 ^a^	1.60 ^a^	1.49 ^a^
HyFlex CM	2.60 ^b^	2.53 ^b^	2.40 ^b^	2.51 ^b^

**Table 2:** Summary score of the debris.

Group	Canal level	Score = 1	Score = 2	Score = 3	Score = 4	Score = 5
Mtwo	Coronal	11	3	1	0	0
	Middle	10	4	1	0	0
	Apical	9	4	2	0	0
Revo-S	Coronal	11	3	1	0	0
	Middle	9	4	2	0	0
	Apical	9	3	3	0	0
HyFlex CM	Coronal	1	4	10	0	0
	Middle	2	3	10	0	0
	Apical	3	3	9	0	0

**Table 3:** Average score of the smear layer for the coronal, middle and apical third of the canals (Values with the same superscript letters were not statistically different at * *P* = 0.05).

Group	Coronal	Middle	Apical	Overall
Mtwo	1.13 ^a^	1.33 ^a^	1.40 ^a^	1.29 ^a^
Revo-S	1.20 ^a^	1.27 ^a^	1.33 ^a^	1.27 ^a^
HyFlex CM	2.33 ^b^	2.60 ^b^	3.27 ^b^	2.73 ^b^

**Table 4:** Summary score of the smear layer.

Group	Canal level	Score = 1	Score = 2	Score = 3	Score = 4	Score = 5
Mtwo	Coronal	13	2	0	0	0
	Middle	11	3	1	0	0
	Apical	11	2	2	0	0
Revo-S	Coronal	12	3	0	0	0
	Middle	12	2	1	0	0
	Apical	12	1	2	0	0
HyFlex CM	Coronal	4	4	5	2	0
	Middle	2	5	5	3	0
	Apical	0	3	7	3	2

**Figure 4:**
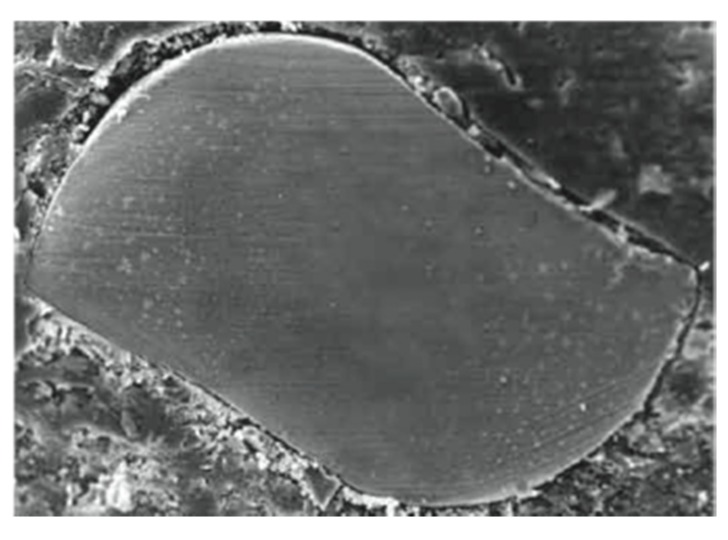
SEM image of Mtwo’s “Italic S” cross section with only two cutting edges.

**Figure 5:**
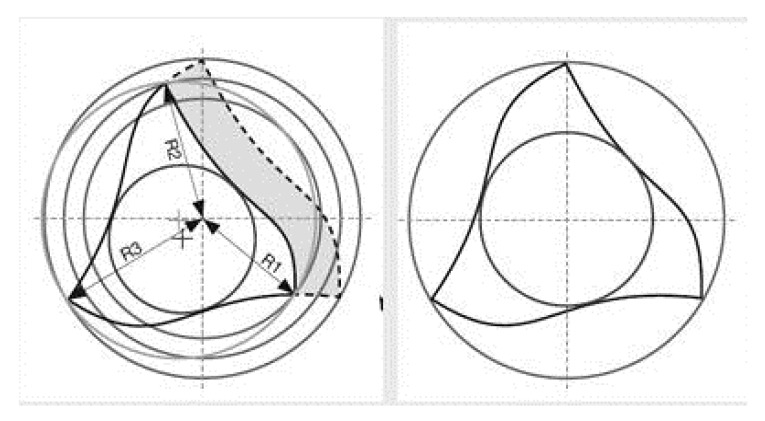
Revo-S section with three cutting edges located on different radiuses.

**Figure 6:**
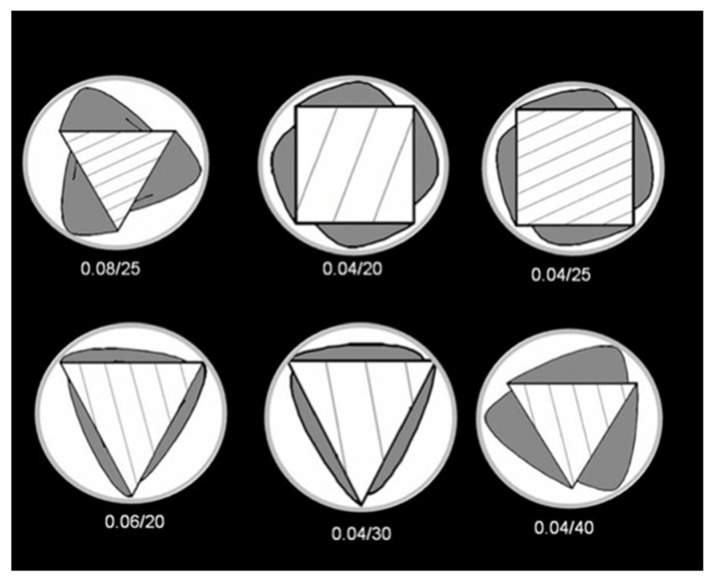
HyFlex sequence: some instruments have triangular cross section with three blades and three flutes, others have quadrangular cross section with four blades and four flutes. Numbers reported below each section indicate the instrument of the sequence.

The null hypothesis of the present study has been rejected. Significant differences were found between the three groups of NiTi rotary instruments. The goal of endodontic treatment is the removal of the debris and smear layer created by instrumentation from the root canal system [1]. During root canal preparation, the action of endodontic instruments produces debris and smear layer, which is compacted along dentinal walls [3]. Its elimination seems to be of great importance, since it could allow NaOCl to penetrate into the dentinal tubules, thus enhancing its bactericidal action [7,8,9]. Moreover, the smear layer may affect the sealing efficiency of root canal filling materials, acting as a physical barrier to sealers [4,5]. All tested instruments were evaluated in accordance with the manufacturers’ direction. All protocol sequences and instruments operative settings were respected: irrigation procedures were standardized for all experimental groups and the same trained operator shaped all root samples. Although it is preferable to use a larger number of samples for endodontic research it has become increasingly difficult to obtain extracted human teeth for laboratory studies in Italy. This study demonstrates that the three NiTi rotary instruments tested produced different dentine surface on root canal walls. SEM analysis revealed that Mtwo and Revo-S rotating files associated to EDTA and NaOCl irrigation leave dentine surfaces substantially free from smear layer. Despite some minor differences all these instruments removed smear layer and debris produced during instrumentation and subsequently dissolved by EDTA. Previous SEM studies investigated the effect of NiTi rotary instruments on dentine and obtained similar results [14,18,19]. The present study also confirmed that the apical third is the area where more debris is still visible under SEM inspection [18]. It is likely that rotating NiTi instruments produced fine dentine particles and debris that were spread and compacted along dentine walls and then partially dissolved by EDTA and removed coronally via flute spaces. Mtwo, thanks to their “italic S” cross section with only two cutting edges (Figure 4), and Revo-S, thanks to their asymmetrical section and three cutting edges located on different radiuses (Figure 5), favorite debris elimination and provided dentinal root walls generally free from smear layer and debris. HyFlex CM instruments are new NiTi rotary instruments with shape memory produced by an innovative methodology (patent pending) that uses a complex heating and cooling treatment that controls the material’s memory. These instruments were made from a specific nickel-titanium alloy that has been claimed to have a lower percent in weight of nickel (52%) [26]. A specific sequence of heat treatments is involved in their manufacturing process leading to a significantly more flexible instrument, measured by Testarelli *et al.* [26] in a standard ISO 3630-1 bending test. The manufacturer claims that they are up to 300% more fatigue-resistant and regain their shape after sterilization [27]. If submitted to excessive resistance or stresses they could be plastically deformed and sterilization in autoclave will result in the instrument regaining its shape [27]. Recently, HyFlex CM size 25, .06 taper instruments were found to be significantly more flexible compared to Profile (Dentsply Tulsa Dental, Tulsa, OK, USA), Hero (MicroMega, Besancon, France) and EndoSequence (Brasseler, Savannah, GA, USA) [26]. Peters *et al.* [27] verified they are bendable and very flexible, with similar torsional resistance compared to instruments made of conventional NiTi alloy; fatigue resistance is much higher and canal preparation ability appear to result in less lower working torque, compared to other conventional rotary instruments. No other information regarding their clinical properties and behavior is known. Shaping ability seems to be effective; however, the subsequent cleanliness provided by the combination with irrigating solutions is not statistically similar to the others sequences. This result probably is related to the non-uniform shape given to the root canal, thus preventing the flushing of debris and smear layer. Peters *et al.* [27] concluded that future experiments about canal shaping ability of HyFlex CM are needed. This first study, with its limitations, showed that even if associated to EDTA and NaOCl irrigating solutions they are not so effective in removing debris produced during endodontic instrumentation. A lot of the smear layer is present along dentinal walls, in apical, middle or coronal portions. This *in vitro* study tested HyFlex CM at room temperature, lower than body temperature. Maybe this could represent a limit, but it has to be demonstrated. A lower temperature could reduce physical properties of their alloy, but is not directly related to their shaping and cleaning capability. It is probably related to their cross section and flutes: some instruments (.02/20, .06/20, .04/30 and .04/40) have triangular cross section with three blades and three flutes, others (.04/20 and .04/25) have quadrangular cross section with four blades and four flutes (Figure 6). The geometry of flutes could be related to low capacity of debris removal and a higher amount of smear layer found along dentinal walls. Two symmetrical blades characterize Mtwo cross-section, while three asymmetrical blades characterize Revo-S cross-section. Both geometries reduce the contact lengths of the blades on the dentinal walls thus reducing the production of debris and smear layer and increase the available volume for irrigating solutions and upward debris elimination. Moreover, the lower number of instruments in Mtwo and Revo-S systems may make a lower production of smear layer easily removable by irrigating solutions.

## 3. Experimental Section

Thirty single-rooted human teeth freshly extracted for periodontal reasons were selected for this study and placed in saline at room temperature immediately after extraction. The inclusion criteria are: morphological similarity, single-canal roots, straight roots, absence of root decay, absence of previous endodontic treatment, root length of at least 13 mm and apical diameter of at least #20. The crown of each tooth was removed at the level of the cementum-enamel junction (CEJ) in order to obtain root segments similar in length. Two longitudinal grooves were prepared on the palatal/lingual and buccal surfaces of each root with a diamond bur used with a high-speed water-cooled handpiece to facilitate vertical splitting with a chisel after canal instrumentation.

### 3.1. Root Canal Instrumentation

The same trained operator prepared all root canals. The root canals were preliminary instrumented using stainless steel #08-10-15 K-files (Maillefer, Konstanz, Germany) to create a glide path and then shaped with three different NiTi rotary instruments:

- (Group A) Mtwo (Sweden Martina, Due Carrare, Padova, Italy);
- (Group B) Revo-S (MicroMega, Besancon, France);
- (Group C) HyFlex CM (Coltene Whaledent AG, Altstatten, Switzerland).

The instruments were used with a digital endodontic engine (Endo Mate DT, NSK, Kanuma, Japan) in clockwise rotation respecting manufacturers’ instructions and protocols. Mtwo protocol requires four files sequence (engine settings: 300 rpm and 2.0 N/cm): 10/.04, 15/.05, 20/.06, 25/.06 and 30/.05, with apical diameter of 0.30 mm and 5% taper finishing preparation. Revo-S protocol requires three files sequence (engine settings: 350 rpm and 3.0 N/cm): SC1, SC2 and SU, with apical diameter of 0.25 mm 6% taper finishing preparation. HyFlex CM protocol requires five files sequence (engine settings: 500 rpm and 2.5 N/cm): .08/25, .04/20, .04/25, .06/20 and .06/25, with apical diameter of 0.25 mm 6% taper finishing preparation. The same trained operator instrumented all the samples. All the roots were randomly assigned to three groups of 10 specimens each. Root canals were irrigated during instrumentation between each file change with 1 mL of 5.25% NaOCl followed by 1 mL of 17% EDTA. After preparation 4 mL of 17% EDTA were left *in situ* for 120 s followed by 1 mL of 5.25% NaOCl for 60 s as final rinse. The same manufacturer (Ogna Laboratori Farmaceutici, Muggiò, Italy) prepared the endodontic irrigating solutions. The irrigating solutions were frequently replaced to maintain their effectiveness. Small endodontic needless (27G Kendall Monoject, Mansfield, MA, USA) allowed us to reach the apical third with the reflux of irrigating solutions. Finally, all the canals were washed with ethanol for 30 s and dried with calibrated paper points (Absorbent Paper Points, Denstply-Maillefer, Konstanz, Germany).

### 3.2. SEM Preparation and Examination

Each sample were dipped in liquid nitrogen immediately after canal preparation and split longitudinally into two halves with a stainless steel chisel. The sections were then prepared for SEM analysis. The sections were then allowed to air-dry overnight in a desiccator at room temperature, sputter-coated with gold and prepared for SEM analysis (EVO MA 10 Carl Zeiss SMT AG, Germany).

SEM observations were obtained at standard magnification of 1000×. Six photomicrographs were taken in three areas (coronal, middle and apical). In a blind manner, three trained operators scored the presence or absence debris and smear layer on the surface of the root canal at the coronal, middle, and apical portion of each canal. All disagreement was resolved by consensus. Hulsmann *et al.* [28] proposed the rating system and the criteria for the scoring are reported following.

Score of the debris:

- Score 1: clean root canal wall, only few small debris particles;
- Score 2: few small agglomerations of debris;
- Score 3: many agglomeration of debris covering less than 50% of the root canal wall;
- Score 4: more than 50% of the root canal wall covered by debris;
- Score 5: complete or nearly complete root canal wall covered by debris.

Score of the smear layer:

- Score 1: no smear layer, orifices of dentinal tubules open;
- Score 2: small amount of smear layer, some dentinal tubules open;
- Score 3: homogenous smear layer covering the root canal wall, only few dentinal tubules open;
- Score 4: complete root canal wall covered by a homogenous smear layer, no open dentinal tubules;
- Score 5: heavy, homogenous smear layer covering the entire root canal wall.

### 3.3. Statistical Analysis

Debris and smear layer scores were separately recorded. Data were analyzed with Kruskal-Wallis test. Mann-Whitney *U* test was performed for post-hoc comparisons. Significance was predetermined for *P* < 0.05.

## 4. Conclusions

More tests with a larger number of samples are needed to fully evaluate advantages and eventually disadvantages of instruments made by new alloy. Results of the present study can leave doubts about their clinical efficacy. Within the limitation of this study, HyFlex CM seem to be not so effective in promoting cleanliness of root canal walls and in removing smear layer from dentine if compared to Mtwo and Revo-S.

## References

[1] Torabinejad M., Walton R.E. (2009). Endodontics: Principles and Practice.

[2] Abou-Rass M., Piccinino M.V. (1982). The effectiveness of four clinical irrigation methods on the removal of root canal debris. Oral Surg. Oral Med. Oral Pathol. Oral Radiol..

[3] Briseño B.M., Wirth R., Hamm G., Standhartinger W. (1992). Efficacy of different irrigation methods and concentrations of root canal irrigation solutions on bacteria in the root canal. Dent. Traumatol..

[4] Kaplan A.E., Picca M., Bonzalez M.I., Macchi R.L., Molgatini S.L. (1999). Antimicrobial effect of six endodontic sealers: An *in vitro* evaluation. Dent. Traumatol..

[5] Mickel A.K., Nguyen T.H., Chogle S. (2003). Antimicrobial activity of endodontic sealers on enterococcus faecalis. J. Endod..

[6] Brown J.I., Doran J.E. (1975). An *in vitro* evaluation of the particle flotation capability of various irrigating solutions. J. Calif. Dent. Assoc..

[7] D’Arcangelo C., Varvara G., de Fazio P. (1999). An evaluation of the action of different root canal irrigants on facoltative aerobic-anaerobic, obligate anaerobic, and microaerophilic bacteria. J. Endod..

[8] Jeansonne M.J., White R.R. (1994). A comparison of 2.0% chlorhexidine gluconate and 5.25% sodium hypochlorite as antimicrobial endodontic irrigants. J. Endod..

[9] Jeansonne J., Batista M., Fraga R., de Uzeda M. (1998). Antibacterial effects of endodontic irrigants on black-pigmented gram-negative anaerobes and facultative bacteria. J. Endod..

[10] Sundqvist G., Figdor D., Persson S., Sjogren U. (1998). Microbiologic analysis of teeth with failed endodontic treatment and the outcome of conservative retreatment. Oral Surg. Oral Med. Oral Pathol. Oral Radiol..

[11] Shabahang S., Pouresmail M., Torabinejad M. (2003). *In vitro* antimicrobial efficacy of MTAD and sodium hypochlorite. J. Endod..

[12] De-Deus G., Garcia-Filho P. (2009). Influence of the NiTi rotary system on the debridement quality of the root canal space. Oral Surg. Oral Med. Oral Pathol. Oral Radiol..

[13] Rodig T., Hulsmann M., Kahlmeier C. (2007). Comparison of root canal preparation with two rotary NiTi instruments: ProFile 0.04 and GT Rotary. Int. Endod. J..

[14] Wadhwani K.K., Tikku A.P., Chandra A., Shakya V.K. (2011). A comparative evaluation of smear layer removal with ethylenediaminetetraacetic acid in different states: A SEM study. Indian J. Dent. Res..

[15] Radcliffe C.E., Potouridou L., Qureshi R., Habahbeh N., Qualtrough A., Worthington H., Drucker D.B. (2004). Antimicrobical activity of varying concentrations of sodium hypochlorite on the endodontic microorganisms *Actinomyces israelii, A. naeslundii, Candida albicans* and *Enterococcus faecalis*. Int. Endod. J..

[16] Dagna A., Arciola C.R., Florindi F., Scribante A., Saino E., Visai L., Poggio C. (2011). *In vitro* evaluation of antimicrobial efficacy of endodontic irrigants. Int. J. Artif. Organs.

[17] Shuping G.B., Orstavik D., Sigurdsson A., Trope M. (2000). Reduction of intracanal bacteria using nickel-titanium rotary instrumentation and various medications. J. Endod..

[18] Foschi F., Nucci C., Montebugnoli L., Breschi L., Malagnino V.A., Prati C. (2004). SEM evaluation of canal wall dentine following use of Mtwo and ProTaper NiTi rotary instruments. Int. Endod. J..

[19] Pérez-Heredia M., Ferrer-Luque C.M., González-Rodríguez M.P. (2006). The effectiveness of different acid irrigating solutions in root canal cleaning after hand and rotary instrumentation. J. Endod..

[20] Yang G., Wu H., Zheng Y., Zhang H., Li H., Zhou X. (2008). Scanning electron microscopic evaluation of debris and smear layer remaining following use of ProTaper and Hero Shaper instruments in combination with NaOCl and EDTA irrigation. Oral Surg. Oral Med. Oral Pathol. Oral Radiol..

[21] Rollison S., Barnett F., Stevens R.H. (2002). Efficacy of bacterial removal from instrumented root canals *in vitro* related to instrumentation technique and size. Oral Surg. Oral Med. Oral Pathol. Oral Radiol..

[22] De Lima Machado M.E., Bichels Sapia L.A., Cai S., Martins G.H., Nabeshima C.K. (2010). Comparison of two rotary systems in root canal preparation regarding disinfection. J. Endod..

[23] Dagna A., Arciola C.R., Visai L., Selan L., Colombo M., Bianchi S., Poggio C. (2012). Antibacterial efficacy of conventional and single-use Ni-Ti endodontic instruments: An *in vitro* microbiological evaluation. Int. J. Artif. Organs.

[24] Economides N., Liolios E., Kolokuris I., Beltes P. (1999). Long-term evaluation of the influence of smear layer removal on the sealing ability of different sealers. J. Endod..

[25] Karagöz-Küçükay I., Bayirli G. (1994). An apical leakage study in the presence and absence of the smear layer. Int. Endod. J..

[26] Testarelli L., Plotino G., Al-Sudani D., Vincenzi V., Giansiracusa A., Grande N.M., Gambarini G. (2011). Bending properties of a new nickel-titanium alloy with a lower percent by weight of nickel. J. Endod..

[27] Peters O.A., Gluskin A.K., Weiss R.A., Han J.T. (2012). An *in vitro* assessment of the physical propeties of novel Hyflex nickel-titanium rotary instruments. Int. Endod. J..

[28] Hulsmann M., Rummelin C., Schafers F. (1997). Root canal cleanliness after preparation with different endodontic handpieces and hand instruments: A comparative SEM investigation. J. Endod..

